# Robotic surgery for bowel endometriosis: a multidisciplinary management of a complex entity

**DOI:** 10.1007/s10151-023-02904-0

**Published:** 2024-02-08

**Authors:** G. N. Piozzi, V. Burea, R. Duhoky, S. Stefan, C. So, D. Wilby, D. Tsepov, J. S. Khan

**Affiliations:** 1 Department of Colorectal Surgery, Portsmouth Hospitals University NHS Trust, Queen Alexandra Hospital, Southwick Hill Road, Cosham, Portsmouth, PO6 3LY UK; 2 The Princess Grace Hospital Robotic Endometriosis Centre, The Harley Street Clinic, HCA Healthcare UK, London, UK; 3 Department of Urology, Portsmouth Hospitals University NHS Trust, Portsmouth, UK; 4 University of Portsmouth, Portsmouth, UK

**Keywords:** Bowel endometriosis, Robotic surgery, Deep infiltrating endometriosis, Multidisciplinary team, Colorectal surgery

## Abstract

**Background:**

Bowel endometriosis impacts quality of life. Treatment requires complex surgical procedures with associated morbidity. Precision approach with robotic surgery leads to organ preservation. Bowel endometriosis requires a multidisciplinary management to improve patient outcomes. This study evaluates perioperative outcomes of bowel endometriosis undergoing multidisciplinary planning and robotic surgery.

**Methods:**

Consecutive cases of multidisciplinary robotic bowel endometriosis procedures (January 2021–December 2022) were evaluated from a prospectively maintained database in a national endometriosis accredited centre. Patients were managed through a multidisciplinary setting including gynaecologists, colorectal robotic surgeons, and other specialists. Dyschezia (menstrual and non-cyclical) and quality of life were assessed pre- and postoperatively (6 months) through validated questionnaires.

**Results:**

Sixty-eight consecutive cases of robotic bowel endometriosis were included. Median age was 35.0 (30.2–42.0) years. Median body mass index was 24.0 (21.0–26.7) kg/m^2^. Procedures performed were 48 (70.6%) shavings, 11 (16.2%) deep shavings, 3 (4.4%) disc excisions, and 6 (8.8%) segmental resections. One (1.5%) patient required temporary stoma. Median operating time was 150 (120–180) min. There were no conversions/return to theatre postoperatively. Median endometriotic nodule size was 25.0 (15.5–40.0) mm. Two (2.9%) patients developed postoperative complications. Median length of postoperative stay was 2 (2–4) days. Median follow-up was 12 (7–17) months. One (1.5%) patient recurred. Median menstrual dyschezia score improved from 5.0 (2.0–8.0) to 1.0 (0.0–5.7). Median non-cyclical dyschezia significantly improved (*p* < 0.001) from 1.0 (0.0–5.7) to 0.0 (0.0–2.0). Median quality of life score improved from 52.5 (35.0–70.0) to 74.5 (60.0–80.0).

**Conclusions:**

Robotic multidisciplinary approach to bowel endometriosis provides good perioperative outcomes with improvement of dyschezia and quality of life.

**Supplementary Information:**

The online version contains supplementary material available at 10.1007/s10151-023-02904-0.

## Introduction

Endometriosis is a chronic inflammatory oestrogen-dependent condition characterized by ectopic endometrial glands and stroma outside the endometrial cavity, usually the pelvis, often associated with severe fibrosis [1, 2]. Endometriosis, affecting 10% of reproductive-age women [3], has three main presentations: peritoneal, ovarian, and deep infiltrating endometriosis (DIE).

DIE (20% of endometriosis) [4] is the most aggressive form, and is characterized by nodules infiltrating more than 5 mm beneath the peritoneal surface of surrounding structures [5]. DIE usually localizes in the posterior compartment and involves the bowel in 3.8–37% of cases [2, 6], with sigmoid and rectum making up 70–90% of them [7]. Bowel DIE is associated with chronic pelvic pain, subfertility, dysmenorrhoea, deep dyspareunia, cyclical bowel or bladder symptoms (dyschezia, bloating, constipation, rectal bleeding, diarrhoea, haematuria), abnormal menstrual bleeding, chronic fatigue, and low back pain, which can negatively affect quality of life (QoL) [8].

Surgery is frequently recommended since medical treatment is often ineffective as a result of fibrosis [9–11]. DIE excision may resolve symptoms and improve QoL but can be associated with recurrence (34%) [12].

Surgical treatment of bowel DIE requires a multidisciplinary approach involving colorectal surgeons [13–15]. Recommendations for surgical treatment of DIE from the Working Group of the European Society for Gynaecological Endoscopy (ESGE), European Society of Human Reproduction and Embryology (ESHRE), and World Endometriosis Society (WES) highlighted the necessity to organize the surgical team, involving other specialists, according to the planned procedure(s). The multidisciplinary team (MDT) should meet in advance before surgery [9]. National Institute for Health and Care Excellence (NICE) Endometriosis: Diagnosis and Management 2017 Guidelines (NG73) advocated to refer women with suspected/confirmed DIE involving bowel, bladder, or ureter to the specialist endometriosis service [16]. The British Society for Gynaecological Endoscopy (BSGE) has established criteria for accreditation of centres for treatment of severe endometriosis: (1) Dedicated consultant-led endometriosis service running within a specialist outpatient clinic; (2) Sufficient workload (≥ 12 cases/year of rectovaginal endometriosis requiring pararectal space dissection); (3) Colorectal surgeon; (4) Other clinicians (urologists, radiologists, and pain management specialists); (5) Data collection (follow-up ≥ 2 years): (6) Endometriosis specialist nurse; (7) Submission of an exemplar surgical video for laparoscopic excision of severe rectovaginal endometriosis [17].

Surgical treatment of bowel DIE depends on the number of lesions, location, depth of infiltration, and extent of bowel lumen involvement/stenosis [9, 18]. Surgical treatment can be conservative (shaving and disc excision) or radical (segmental resections) [19].

A minimally invasive approach is the gold standard since it reduces blood loss, decreases pain, improves cosmesis, improves recovery, reduces hospital stay, improves fertility, and reduces postoperative morbidity compared to laparotomy [20–22]. However, laparoscopy with standard straight instruments can be challenging for bowel DIE because of narrow operating field, restricted motion, and fibrosis.

The da Vinci (Intuitive Surgical, Sunnyvale, CA, USA) robotic platform was specifically developed to compensate for some deficiencies of laparoscopy by improving dexterity, surgical precision, and view [23, 24]. Robotics allows for stable and fine dissection in challenging districts (i.e. narrow deep pelvic cavity) providing better surgical outcomes for complex pelvic techniques [25]. Moreover, robotics facilitates the translation of open urological reconstructive procedures, such as ureteric reimplantation and partial cystectomy, to the minimal access route without compromising functional outcomes. The technical advantage should be carefully considered since DIE affects especially young fertile women. The benefits of using a robotic approach to treat severe bowel DIE are currently under investigation [14, 26–28].

This study aimed to evaluate the perioperative outcomes of patients with severe bowel DIE undergoing multidisciplinary surgical treatment (gynaecologist and colorectal surgeon) with a robotic approach.

## Methods

### Study population

This study retrospectively evaluated a consecutive series of multidisciplinary robotic resections for bowel DIE performed between January 2021 (opening of the Robotic Endometriosis Centre at The Princess Grace Hospital) and December 2022. Data were extracted from a prospectively maintained endometriosis database in a BSGE-accredited endometriosis centre.

Patients provided informed consent for prospective anonymized data collection for the BSGE national registry for research purpose (accreditation requirement).

The primary aim was to report perioperative outcomes of patients undergoing multidisciplinary resections. The secondary aim was to report dyschezia and QoL pre- and postoperatively (6 months).

Inclusion criteria were (1) robotic approach; (2) pararectal space dissection; (3) multidisciplinary approach involving colorectal surgeons with/without urologist; (4) endometriosis on histology; (5) age ≥ 18 years.

The was one exclusion criterion: no specialized colorectal surgeon.

### MDT discussion and planning

**Fig. 1:**
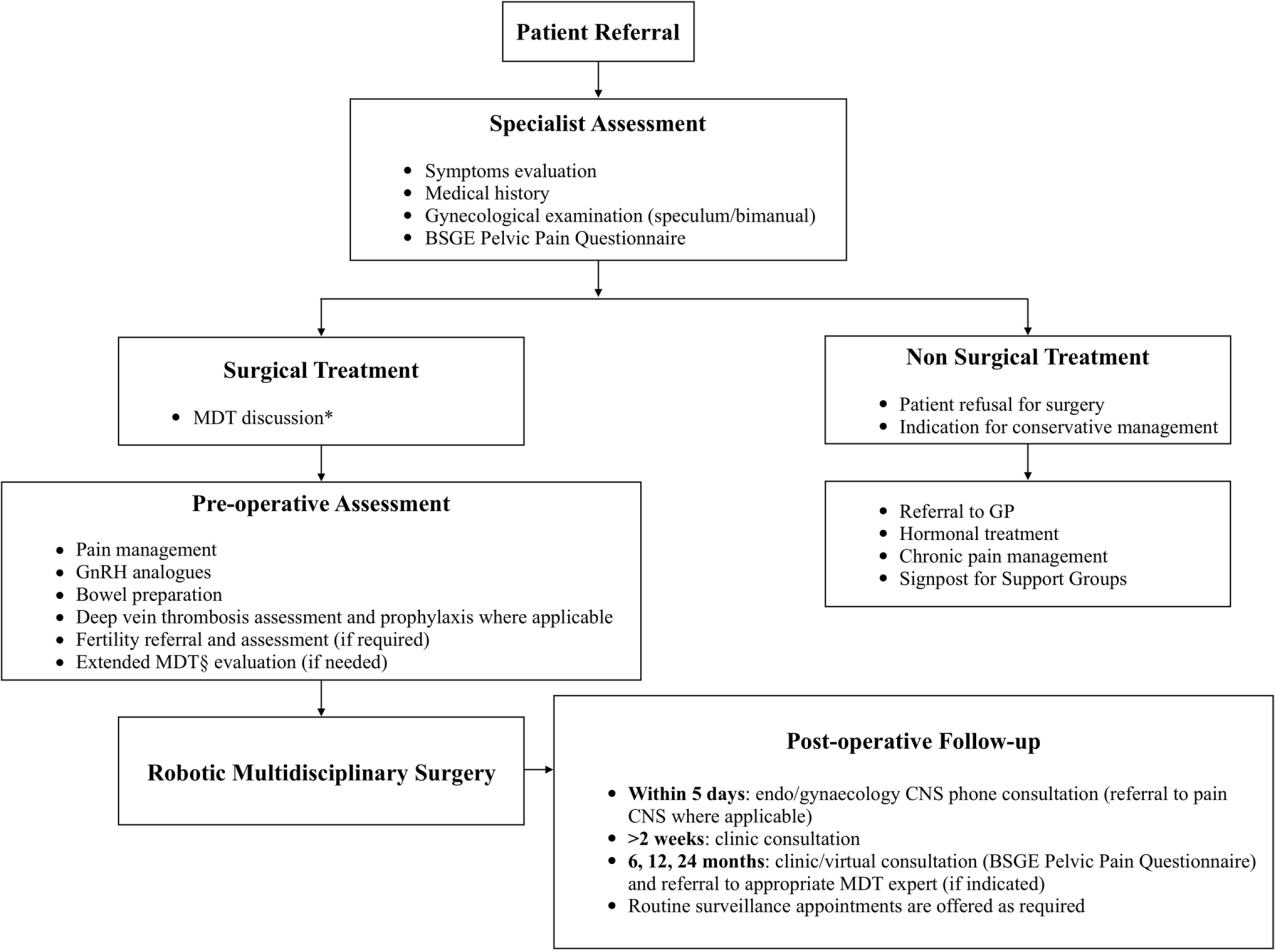
Perioperative endometriosis management pathway. MDT*: includes gynaecologists, colorectal surgeons, urological robotic surgeons, anaesthetists, radiologists, nurse specialist (gynaecological and pain management), and database administrators. MDT§: includes general robotic surgeons, cardiothoracic surgeons, gastroenterologists, haematologists, psychologists, psychiatrists, psychosexual therapists, stoma nurses, pain consultants, fertility specialists, women’s health physiotherapy team, and dieticians. *BSGE* British Society for Gynaecological Endoscopy, *CNS* clinical nurse specialist, *GnRH* gonadotropin-releasing hormone, *GP* general practitioner, *MDT* multidisciplinary team

Preoperative staging workup included (Fig. 1) collection of symptoms, medical history, gynaecological examination (speculum/bimanual), and BSGE pelvic pain questionnaire completion. Faecal occult blood test, pelvic and transvaginal ultrasound, abdominopelvic magnetic resonance imaging, and colonoscopy were performed if indicated.

Surgery was offered when symptoms were unresponsive to medical treatment and was carefully discussed at an MDT meeting and with the patient. The MDT (including gynaecologists, colorectal surgeons, urologists, cardiothoracic surgeons, radiologists, gastroenterologists, psychologists, psychiatrists, psychosexual therapists, stoma nurses, women’s health physiotherapy team, nurse specialist (gynaecological and pain management), dieticians, database administrators and any other extended MDT members, if required) prepared a patient-tailored surgical plan. Fertility referral, GnRH analogues, and pain management were recommended if needed.

### Surgical technique

Surgeries were performed with the da Vinci Xi® platform with dual console by an MDT including gynaecologist, colorectal surgeon, and urologist with extensive expertise in minimally invasive surgery.

Mechanical bowel preparation and deep vein thrombosis prophylaxis were performed.

Lloyd-Davies position (22° head-down) was adopted. Pneumoperitoneum (10 mmHg) was obtained through Veress needle technique.

**Fig. 2:**
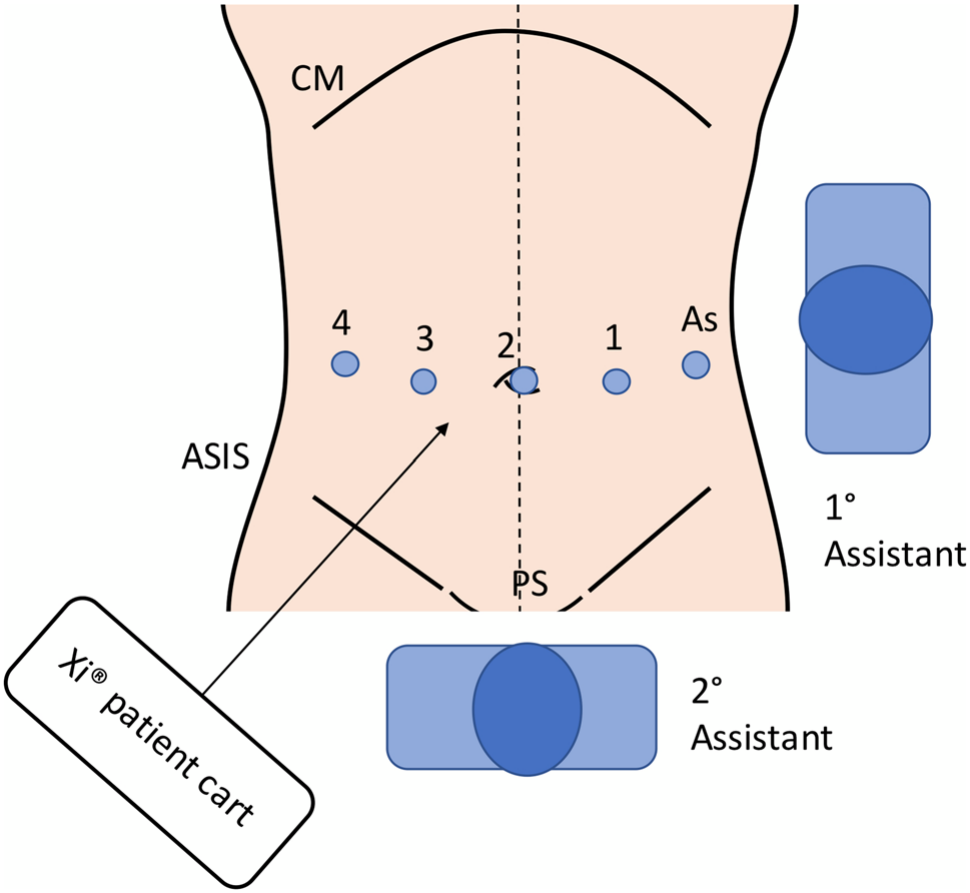
Port and patient cart placement asset. *As* assistant port, *ASIS* anterior superior iliac spine, *CM* costal margin, *PS* pubic symphysis

A five-port transverse approach (Fig. 2) was adopted with four robotic 8-mm trocars and one 8-mm AirSeal® access port (ConMed, Utica, NY, USA; used by the assistant for suction or traction). A single-docking fully robotic approach with a two right-hand setting was used: arm 1 (bipolar forceps), arm 2 (30° endoscope), arm 3 (monopolar scissors), arm 4 (Cadiere forceps). Second assistant used VCare® Plus (ConMed, Utica, NY, USA) intrauterine manipulator.

dV3 monopolar and dV1 bipolar system (Intuitive Surgical, Inc., Sunnyvale, CA, USA) were used for energy devices. Monopolar setting was 3, cut auto (180 W max); 3, coag swift (150 W max). Bipolar setting was 3, auto (80 W max).

A gynaecologist performed adhesiolysis, drainage, and stripping of endometriomas. Resection varied according to disease location, extension, and patient’s fertility desire. Then, gynaecologists and colorectal surgeon together performed nodules resection with shaving, disc excision, or segmental resection according to number of lesions, location, contiguity, size, depth of infiltration, distance from the anal verge, and circumferential involvement. All dissections were performed with nerve-sparing technique. Shavings were performed with monopolar cut function and grouped in superficial shaving (serosa and outer third muscularis layer excision) and deep shaving (serosa with middle third muscularis layer excision with interrupted suture repair in single layer). For disc excision, the bowel wall including the nodule was resected with a transanal circular stapler. Segmental resection was performed with low ligation of the superior rectal artery at the level of DIE. All colorectal anastomoses were evaluated with a triple assessment using fluorescence (3 ml indocyanine green infusion), air leak, and endoscopic evaluation (“Portsmouth protocol”) [29].

Conversion was defined as unintended extension of the suprapubic extraction site incision.

### Postoperative follow-up

All specimens underwent pathological evaluation. Complications were assessed according to Clavien-Dindo’s classification [30].

Postoperative continuous hormone therapy was recommended to patients with no pregnancy intention to reduce postoperative recurrences.

Figure 1 reports the postoperative follow-up. Referral to the appropriate MDT expert (colorectal, urologist, HPB surgeon, pain management, gastroenterologist, dietician, other) was made when clinically indicated.

STROBE statement for cohort studies was followed [31].

### Pain questionnaires

The BSGE Pelvic Pain Questionnaire (Supplementary file 1) was completed in person at baseline (preoperatively) and through email at 6 months’ follow-up. Menstrual dyschezia was calculated from responses to question “pain opening bowels during period”, while non-cyclical dyschezia was calculated from responses to question “pain opening bowels at other times”. QoL scores were evaluated through Likert scale (Q7).

### Statistical analysis

Patient characteristics were summarized using basic descriptive statistics. Continuous variables were presented as median (interquartile range, IQR) and compared using Mann–Whitney *U* test. Categorical variables were expressed as proportions and analyzed using chi-squared or Fisher’s exact test. Statistical analysis was performed using IBM SPSS Statistics for Macintosh, version 28 (IBM Corp., Armonk, NY, USA). Confidence intervals were estimated at 95%, and significance level set at *p* = 0.05.

## Results

### Patient characteristics

Sixty-eight patients were enrolled. Series characteristics are listed in Table 1. Median age was 35.0 (30.2–42.0) years. Median BMI was 24.0 (21.0–26.7) kg/m^2^ with six obesity class I, two class II, and three class III patients. ASA score II was predominant (*n* = 64, 95.6%). All patients had bowel DIE (grade severe/IV), unresponsive to medical therapy, requiring pararectal space dissection.

**Table 1:** Series characteristics

	*n* = 68
Age, years	35.0 (30.2–42.0)
BMI, kg/m^2^	24.0 (21.0–26.7)
ASA	
I	3 (4.4%)
II	65 (95.6%)
Location nodules	
Rectovaginal	43 (63.2%)
Rectosigmoid	14 (20.6%)
Rectovaginal and rectosigmoid	10 (14.7%)
Rectovaginal, caecum, and ileum	1 (1.5%)
Operating time, min	150 (120–180)
Estimated blood loss, ml	50 (50–100)
Number of nodules removed	2 (1–3)
Size max, mm	25.0 (15.5–40.0)
Involvement of muscularis propria	28 (41.2%)
Disease-free margin	68 (100%)
Malignancy	0
Type of resection	
Shave	48 (70.6%)
Deep shave	11 (16.2%)
Disc resection	3 (4.4%)
Segmental resection	6 (8.8%)
Stoma	1 (1.5%)
LOS, days	2 (2–4)
Complications	2 (2.9%)
ICU admission	0 (0%)
Mortality	0
Histological confirmation	68 (100%)
Follow-up, months	12 (7–17)

Data are reported as median (interquartile range) or *n* (%)

*ASA* American Society of Anestesiologists, *BMI* body mass index, *ICU* intensive care unit, *LOS* length of postoperative stay

Bowel DIE’s location was rectovaginal (*n* = 43, 63.2%); rectosigmoid (*n* = 14, 20.6%); rectovaginal and rectosigmoid (*n* = 10, 14.7%); rectovaginal, caecum, and small bowel (*n* = 1, 1.5%).

### Operative outcomes

All bowel procedures were performed as a joint case by the gynaecologist and colorectal surgeon. Six (8.8%) patients required a segmental resection: one for a 90-mm lesion (12 cm from the anal verge) together with an ileocecal resection for caecum endometriosis; one for a 150-mm and another for a 140-mm large sigmoid nodule; three other patients for a 40, 80, and 145-mm full thickness rectal mural infiltration nodule.

Three cases (4.4%) were treated with transanal stapled disc resection. One patient had rectovaginal nodules infiltrating down to the mucosa, the disc resection was 5 cm long and included all three nodules; one patient had one 32-mm nodule infiltrating the sigmoid colon; one had both a disc resection for two rectovaginal mural nodes and a shaving for a superficial 6-mm node.

Deep and superficial shaves were successfully performed in a total of 11 (16.2%) and 48 (70.6%) patients, respectively.

Four patients (5.9%) required an appendectomy (one performed during ileocecal resection) for endometrial infiltration. One patient who underwent deep shaving and suturing had positive air leak test twice during surgical repair and therefore a diverting ileostomy was created and reversed after 6 months.

Median operating time was 150 (120–180) min with two patients having a procedure 240 min long (both segmental resections). Median operating time was significantly different between surgical procedures: 120 (120–150) min for superficial shaving, 180 (150–180) min for deep shavings, 180 (180–180) min for disc resections, 195 (172–240) min for segmental resections (*p* < 0.001).

Median estimated blood loss was 50 (50–100) ml. There were no conversions to laparoscopy or open surgery.

### Pathological results

A median of 2 (1–3) nodules were removed per procedure with a median nodule size (maximum diameter) of 25.0 (15.5–40.0) mm.

Median nodular maximum diameter was significantly different between surgical procedures: 23 (15–33) mm for shaving, 25 (19–30) mm for deep shavings, 25 (15–32) mm for disc resections, and 115 (70–146) mm for segmental resections (*p* = 0.037).

Muscularis mucosa was involved in 28 (41.2%) cases. All resections had endometriosis-free margins. Endometriosis was confirmed in all cases with no evidence of malignancies.

### Postoperative outcomes and follow-up

Two patients (2.9%) developed postoperative complications. One had a pelvic haematoma requiring a blood transfusion (grade II). This was the only patient requiring blood transfusion either intra- and/or postoperatively. One was a grade III ileus associated with pelvic collection requiring a pelvic drain. None of the patients required perioperative reoperation. Median postoperative stay was 2 (2–4) days. Median follow-up was 12 (7–17) months. All patients are in active follow-up.

Questionnaires were answered postoperatively by 63 (92.6%) patients. Median menstrual dyschezia improved from 5.0 (2.0–8.0) to 1.0 (0.0–5.7) and QoL increased from 52.5 (35.0–70.0) to 74.5 (60.0–80.0), at preoperative and 6 months’ follow-up, respectively (Table 2). Only median non-cyclical dyschezia significantly improved (*p* < 0.001) from 1.0 (0.0–5.7) to 0.0 (0.0–2.0) during the same follow-up.

**Table 2:** Dyschezia and quality of life (QoL) scores pre- and postoperatively at 6 months’ follow-up

	Preoperative	Postoperative 6 months	*p*
Menstrual dyschezia	5.0 (2.0–8.0)	1.0 (0.0–5.7)	0.709
Non-cyclical dyschezia	1.0 (0.0–5.7)	0.0 (0.0–2.0)	< 0.001
QoL	52.5 (35.0–70.0)	74.5 (60.0–80.0)	0.099

The data was calculated from the British Society for Gynaecological Endoscopy (BSGE) Pelvic Pain Questionnaires (Supplementary file 1). Data are reported as median (interquartile range)

One patient (1.5%) who received a shaving for a 20-mm rectovaginal nodule developed a recurrence after 3 months requiring a segmental resection with anastomosis 8 cm from the anal verge. The patient desired pregnancy after primary surgery and refused postoperative hormonal suppression.

## Discussion

A robotic multidisciplinary approach to severe bowel DIE provided good perioperative outcomes and improved dyschezia and QoL in the current series. A robotic approach, by enhancing the rates of successful shavings, provides high rates of organ preservation for severe bowel DIE and limits segmental resection only to extreme cases with deeper involvement and greater nodule diameter.

Surgical strategy to bowel DIE shifted from segmental resections to organ preservation [2, 6]. Bowel DIE requires careful patient-tailored surgical strategy; therefore, pre- and intraoperative MDT planning is crucial. Conservative treatments allow one to preserve bowel anatomy and function, and are associated with lower complication rates [32, 33], but could be at risk for higher rate of recurrences [2], even if not always confirmed [33].

A systematic review on 3079 patients (1.7% undergoing a robotic approach) showed significant increase in grade IIIb complications from 5.5% for shavings, to 7.5% for disc excision, and up to 11.8% for segmental resections (*p* < 0.001) [34], later confirmed by a comparative cohort study [6]. Segmental resections are associated with higher risk of rectovaginal fistulas, vascular/nervous injury, anastomotic stenosis, leak, stoma creation, voiding dysfunction, and/or low anterior resection syndrome [2, 6, 35] and are not associated with more long-lasting symptoms improvement [36]. Indeed, higher complications are not only related to the colorectal procedure itself but also to disease severity. For these reasons organ preservation should be attempted and indicated where possible [6]. Abo et al. suggest that in case of nodules with similar characteristics, a personalized management is necessary with a more radical approach for young nullipara with pregnancy intention (delay for recurrence is long), and a more conservative approach for older patients with no pregnancy intention (delay until menopause is short) [6].

The present study reported a very low rate of complications (2.9%) which could be due to our limited indication of segmental resections and therefore higher number of organ preservation procedures, to the expertise of the gynaecologist (> 300 procedures) and colorectal surgeon (> 700 procedures), to the relatively limited number of cases in this series, and to the MDT planning. The MDT fulfilled the hospital and surgeon volume’s criteria suggesting at least 20 procedures per centre per year and at least 8–13 procedures per surgeon per year to decrease surgical complications risk [37].

However, organ preservation is not without risks since shavings were reported being associated with higher rates of endometriosis recurrence compared to disc excision (OR 3.83, *p* = 0.01) and segmental resection (OR 5.54, *p* = 0.001) [38]. This could be consequent to microscopic residual disease. Although the present study included only endometriosis-free margins, one patient (1.5%) undergoing shaving developed a recurrence requiring a segmental resection. Longer follow-up is needed since the literature reports rates as high as 50% after 5-year follow-up [38].

Robotics through better view and dexterity may increase the rate of organ preservation even for more advanced bowel DIE. The LAROSE trial, which is the only randomized trial comparing robotic and laparoscopic approach to endometriosis, showed no difference on operative time, blood loss, intra-/postoperative complications or conversion but specifically excluded patients with bowel resections [26]. Therefore, there is still very limited data on the robotic approach for bowel DIE, especially grade IV.

A recent prospective cohort study comparing robotic and laparoscopic bowel endometriosis (all grades) showed significantly longer total operative time (208 ± 90 vs 169 ± 81, *p* = 0.01) and higher free-margin resections (90.9% vs 76.2%, *p* = 0.01) for robotics with no difference in complications [27]. This study may confirm robotics’ better visualization rate, which was reported to be 2.36 times better than laparoscopy, allowing for theoretical greater disease clearance [39]. When evaluating conservative and radical resections, operative time was different between approaches only for the conservative group and similar for segmental resections [27]. However, when comparing robotic vs laparoscopic disc excision and segmental resections, the overall complication rate was significantly higher for laparoscopy (2.3% vs 14.3%,* p* = 0.04) showing a benefit from robotics for more advanced disease [27]. The authors reported that robotics allowed intraoperative conversion of planned segmental resection to disc excision in five patients [27], reaffirming the significant role on intraoperative re-evaluation and successful surgical strategy change.

Ferrier et al. [27] showed no advantage over conventional laparoscopy when performing robotic shaving, but Hiltunen et al. showed higher rates of shaving performed through a robotic approach [40]. The robotic platform may increase the rate of successful shaving, allowing also deep shaves up to the submucosal layer without affecting perioperative outcomes as seen in the present study (low rate of segmental resections) [40]. In the present series shavings and disc excision had similar nodular diameter showing that shavings can also be indicated for larger and more advanced lesions than previously reported [28]. Segmental resections can be safely indicated specifically for large severe bowel DIE (median of 115 (70–146) mm in the present series).

A robotic approach may be advantageous over laparoscopy because it facilitates adhesiolysis (challenging in severe bowel DIE), facilitates organ preservation, and allows for optimization of disease visualization. The transverse port placement at the umbilicus (Fig. 2) improves view and technique for both gynaecologist and colorectal surgeon, optimizing exploration and manipulation of the lower bowel for disease identification and clearance.

The present study reported improvement in dyschezia and QoL after surgery, even after organ preservation. Although only non-cyclical dyschezia improved significantly, to evaluate menstrual dyschezia we would require a larger series since we may be encountering a survey ceiling effect. Dyschezia is poorly reported in the literature as a result of lack of validated questionnaires, poor response rate, and long follow-up [41]. Soto et al. suggested that surgery provides improvement in dysmenorrhoea, chronic non-cyclical pain, pain with bowel movements, and lower back pain in patients with bowel DIE [41]. Hiltunen et al. reported improvement of symptoms after surgery; however, their questionnaire was not validated [40].

This study has some limitations. First, despite being the most extensive series of robotic multidisciplinary cases for robotic severe (grade IV) bowel DIE, the series is still limited to provide extensive results. Second, this series included only ASA I and II patients; therefore, further studies are needed to confirm the results in higher-risk patients. Third, the short/mid-term follow-up does not allow one to obtain extensive data on endometriosis recurrence after organ preservation; however, the prospective data collection makes the database quality robust. Fourth, this study was performed by experienced robotic surgeons, well passed their learning curve, in a high-volume centre accredited by BSGE which affects the generalizability of the results to all institutions.

This study has several strengths. First, it included only cases of pathologically confirmed bowel endometriosis. Second, this is the widest series on robotic bowel DIE (grade IV) submitted to organ preservation. Third, this shows both superficial and deep shavings. Lastly, dyschezia and QoL were assessed through a standardized and validated questionnaire from BSGE.

Future studies on larger series with longer follow-up are needed to confirm the results and to demonstrate long-term function.

## Conclusion

A robotic MDT approach to severe bowel DIE provides good perioperative outcomes and improves dyschezia and QoL.

## Supplementary Information

Below is the link to the electronic supplementary material.

Supplementary file1 (PDF 247 KB)

## Data Availability

The datasets used or analysed during the current study are available from the corresponding author upon reasonable request.
