# Supplementary material for: Robotic surgery for bowel endometriosis: a multidisciplinary management of a complex entity
Source: Tech Coloproctol. 2024 Feb 8;28(1):31. doi: 10.1007/s10151-023-02904-0 (PMC10853297; doi:10.1007/s10151-023-02904-0)
Supplement: Supplementary file 1 — Supplementary file1 (PDF 247 KB) [file 10151_2023_2904_MOESM1_ESM.pdf]

[Hospital logo here]

Patient label

Centre:
Date of clinic attendance:
Referral: Self/GP/Consultant/website/other
Private patient: YES/NO

BSGE Pelvic Pain Questionnaire

This questionnaire has been designed to help us understand your problems and to find the most appropriate treatments. It may also help you to formulate your thoughts on your symptoms and the way in which they can affect your quality of life. It is important that you answer as many of the questions as you are able. If you find any of them awkward to answer, please leave them blank and we may discuss them at your consultation should you wish.

Please be assured that the information you provide will be kept confidential, in accordance with Data Protection legislation and entered onto a central database together with the results of clinical examination and any tests that you may have. The findings and results of any surgical intervention that you may have will be recorded and assessed, as will your responses from follow up questionnaires.

The anonymous information collected on all patients will be used for research and study into the treatment of endometriosis, and may be published in medical journals or presented at medical scientific meetings.

If you do not understand any of the questions, particularly about previous treatment, please leave these blank and raise the questions when you are seen in the clinic.

Please sign below to confirm that you are happy for the information that you provide to be included on the database. We will be collecting information about you throughout your treatment.

Signature ……………………………………… NAME (Print) …………………………………. Date ……………………………

In some cases it is easier to send you follow up questionnaires by email. Don't worry, we will only send emails when you are due a follow up questionnaire, so this will be a maximum of three questionnaires over two years. If you give consent to receive email questionnaires, please complete the details below:

I consent to email contact.

Email address: ____

Signature ….……………………………………

BACKGROUND DETAILS

Smoking: Current Smoker ☐ Ex Smoker ☐ Never smoked ☐

What is your Height? ________________ Metres

What is your current weight? ________________ Kilograms

1. GENERAL QUESTION ABOUT YOUR PAIN

Over the course of your **current normal menstrual cycle**, which of the following symptoms do you experience? Please tick yes or no to show whether you experience symptom during a normal cycle, and then if you have experienced the symptom, circle a score from 1 to 10 to indicate how slight or severe it usually is. (NOTE: N/A denotes 'no period')

**Pre-menstrual pain** (pain before periods)										Experienced YES ☐ NO ☐ N/A ☐<br>Score 0
Experienced slightly										Experienced severely
1	2	3	4	5	6	7	8	9	10	

**Menstrual pain** (pain during periods)										Experienced YES ☐ NO ☐ N/A ☐<br>Score 0
Experienced slightly										Experienced severely
1	2	3	4	5	6	7	8	9	10	

**Non-cyclical pelvic pain** (pain throughout the month)										Experienced YES ☐ NO ☐<br>Score 0
Experienced slightly										Experienced severely
1	2	3	4	5	6	7	8	9	10	

**Pain during sexual intercourse**										Experienced YES ☐ NO ☐ N/A ☐<br>Score 0
Experienced slightly										Experienced severely
1	2	3	4	5	6	7	8	9	10	

**Pain opening bowels during period**										Experienced YES ☐ NO ☐ N/A ☐<br>Score 0
Experienced slightly										Experienced severely
1	2	3	4	5	6	7	8	9	10	

**Pain opening bowels at other times**										Experienced YES ☐ NO ☐ N/A ☐<br>Score 0
Experienced slightly										Experienced severely
1	2	3	4	5	6	7	8	9	10	

**Lower back pain**										Experienced YES ☐ NO ☐<br>Score 0
Experienced slightly										Experienced severely
1	2	3	4	5	6	7	8	9	10	

**Bladder pain or pain passing urine**										Experienced YES ☐ NO ☐ Score 0
Experienced slightly										Experienced severely
1	2	3	4	5	6	7	8	9	10	

**Do you have difficulty emptying your bladder?**										Experienced YES ☐ NO ☐ Score 0
Experienced slightly										Experienced severely
1	2	3	4	5	6	7	8	9	10	

2. **Information about Bowel function**
(NOTE: N/A is to be used if you have a stoma)

Do you have frequent bowel movements?

Never ☐ a little of the time ☐ some of the time ☐ most of the time ☐ all of the time ☐ N/A ☐

Do you have urgent bowel movements?

Never ☐ a little of the time ☐ some of the time ☐ most of the time ☐ all of the time ☐ N/A ☐

Do you have sensation on incomplete emptying of the bowel?

Never ☐ a little of the time ☐ some of the time ☐ most of the time ☐ all of the time ☐ N/A ☐

Do you have constipation?

Never ☐ a little of the time ☐ some of the time ☐ most of the time ☐ all of the time ☐ N/A ☐

Have you been troubled by blood in the stool around the same time as your period?

Never ☐ a little of the time ☐ some of the time ☐ most of the time ☐ all of the time ☐
Not applicable as I don't have periods ☐

3. **Medical Therapy**

Are you currently taking any of the following treatments?
Please tick to indicate your use.

Oral contraceptive pill **YES** ☐ **NO** ☐

Mirena IUS (hormone containing coil) **YES** ☐ **NO** ☐

GnRH Analouges **YES** ☐ **NO** ☐
E.g. Goserelin, Buserelin, Lupron, Naferelin

GnRH Analouges + oestrogens (HRT) **YES** ☐ **NO** ☐

Progestogens **YES** ☐ **NO** ☐
E.g. Primolut, Duphaston, Provera

**Aromatase inhibitors**	**YES ☐**	**NO ☐**
**Hormone replacement**	**YES ☐**	**NO ☐**

4. <u>Fertility</u>

Are you currently trying to get pregnant?

**No**	☐
**Yes, been trying for less than 18 months**	☐
**Yes, been trying for more than 18 months**	☐

**Are you currently pregnant?**	**YES ☐**	**NO ☐**

5. <u>Do you take any of the following painkillers</u>

**Paracetamol**	**YES ☐**	**NO ☐**
**NSAID anti-inflammatories**<br>*E.g. Ibuprofen, Diclofenac*	**YES ☐**	**NO ☐**
**Opiates**<br>*E.g. Tramadol, DF118*	**YES ☐**	**NO ☐**

6. Have you ever had previous surgery for endometriosis

**Have you had your endometriosis surgically treated before today?**	**YES ☐**	**NO ☐**
**Have you had an ovary removed?**	**YES ☐**	**NO ☐**
**Have you had both ovaries removed?**	**YES ☐**	**NO ☐**
**Have you had a hysterectomy?**	**YES ☐**	**NO ☐**

7. Questions about your health in general

The following questions refer to how you feel about your health in general **TODAY.** They form part of a standard set of questions relating to quality of life and therefore some may not seem particularly relevant to you. However, please try to answer ALL questions.

Please score how good or bad your health is **TODAY.** The best health state you can imagine is marked 100 and the worst health state you can imagine is marked 0.

(Please place a line on the scale between 1 and 100 according to how you feel)

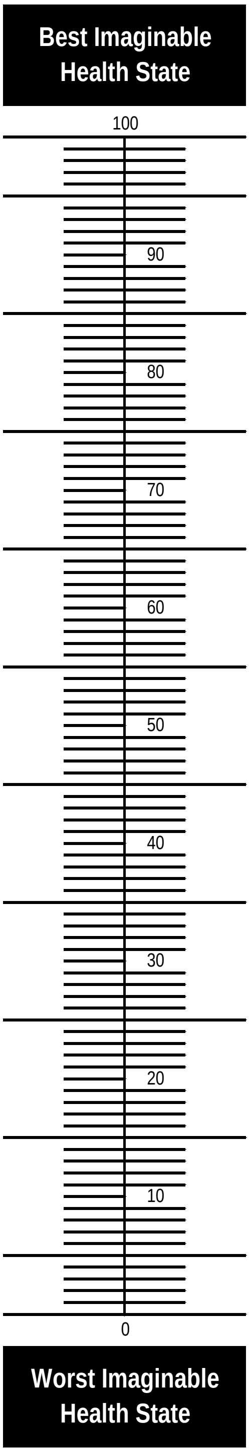

BSGE-ECD PPQ Jan15

8. **Please indicate which statements best describe your health state TODAY**

Usual Activities (e.g. work, study, housework, family or leisure activities)

I have no problems with performing my usual activities ☐

I have some problems with performing my usual activities ☐

I am unable to perform my usual activities ☐

Pain/Discomfort

I have no pain or discomfort ☐

I have moderate pain or discomfort ☐

I have extreme pain or discomfort ☐

Anxiety/Depression

I am not anxious or depressed ☐

I am moderately anxious or depressed ☐

I am extremely anxious or depressed ☐

Mobility

I have no problems in walking about ☐

I have some problems in walking about ☐

I am confined to bed ☐

Self-Care

I have no problems with self-care ☐

I have some problems washing or dressing myself ☐

I am unable to wash or dress myself ☐

Thank you very much for completing this questionnaire.

We would like to reassure you again that all the answers will be treated in the strictest confidence.
